# Protective factors of marital stability in long-term marriage globally: a systematic review

**DOI:** 10.4178/epih.e2019023

**Published:** 2019-06-15

**Authors:** Reza Karimi, Maryam Bakhtiyari, Abbas Masjedi Arani

**Affiliations:** Department of Clinical Psychology, School of Medicine, Shahid Beheshti University of Medical Sciences, Tehran, Iran

**Keywords:** Long-term marriage, Protective factors, Marital stability, Divorce, Systematic review

## Abstract

**OBJECTIVES:**

In recent decades, due to the high prevalence of divorce in numerous countries and the detrimental aftermath thereof, it has become increasingly important to study the components of marital stability. The current study explored fundamental protective factors in long-term marriage through a systematic review.

**METHODS:**

Searches for relevant publications were conducted in Embase, Web of Science, PubMed, Scopus, Science Direct, Magiran, and Scientific Information Database from their inception through January 30, 2019. Through the keyword search, 1,706 articles were found, of which 25 articles remained after screening based on the eligibility criteria.

**RESULTS:**

The extracted protective factors associated with marital stability in long-term marriage were classified as interpersonal and intrapersonal. Notable extracted factors included spirituality and religion, commitment, sexual relationship, communication, children, love and attachment, intimacy, and conflict resolution approach. These findings show that some aspects of relationships, such as commitment, act to preserve the pillars of marriage in critical situations, while other aspects, such as intimacy, help to construct marital identity and satisfaction.

**CONCLUSIONS:**

The identified components of marital stability are structures that enhance a couple’s identity and sense of togetherness. Identifying the specific aspects of marital relationships that contribute to marital stability may help specialists and researchers to target specific types of marital interaction that may enhance the happiness and longevity of relationships, thereby preventing avoidable divorces.

## INTRODUCTION

In the present era, personal and intimate relationships have been established to have positive effects on physical and psychological health in older adults, and most of our perceptions about these linkages come from findings based on long-term marriage [1]. In the past century, although many couples have chosen to stay together in long-term marriages for at least 40 years [2], divorce and separation rates have steadily increased and divorce has become a feasible choice for most couples [3]. Notwithstanding, most existing research in the literature on marriage has focused on the early [4] and middle years of marriages [5], whereas the literature on long-term marriages is sparse [6].

Researchers have investigated a wide factors related to long-term marriage, including attitudes towards marital relations [7]; religion [8-10]; the role of children [2]; love, commitment, and intimacy [11]; gender [12]; communication and conflict resolution [13]; support [14]; attachment and loyalty [15]; and role division [16].

Both older and more recent studies have suggested that the negative components of marital relationships tend to be more closely associated with marital longevity than do the positive components of the physical and psychological well-being of couples [17-21], while many other studies have reported that positive aspects of relationships protect marital stability [2,7,11]. Furthermore, still other research has suggested that combinations of negative and positive factors in a relationship contribute to couples staying together in long-lasting marriages [22,23].

Furthermore, in previous studies, inconsistent findings have been reported regarding gender differences in the protective factors affecting marital success. Some studies have found considerable resemblances in what men and women consider exigent to marital success [24] whereas other studies have found more differences than similarities [25,26].

Inconsistent findings have been reported in the literature regarding whether negative or positive aspects of marital relationships are associated with marital length, and inconsistencies exist with regard to gender differences in these issues. Additionally, in previous studies, the protective factors involved in long-term marriage have been reported to include a broad range of components. Considering these gaps, the purpose of the current study was to shed light on the diverse trajectories of the ingredients of long-term marriages and to explore the important elements that protect marital stability among couples in long-term marriages through a review of all relevant qualitative and quantitative studies.

## MATERIALS AND METHODS

### Search strategy

**Figure 1.**
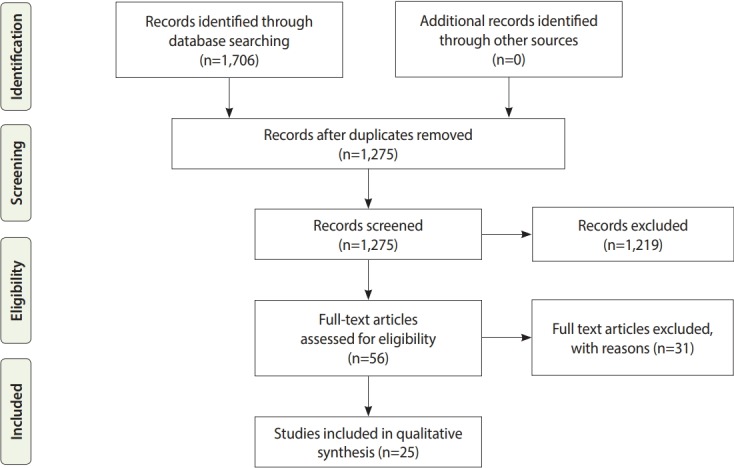
Flow chart for reviewing the literature and retrieval process.

We undertook a systematic search in Persian and English electronic databases. The first step in the strategy entailed a limited search of publications in MEDLINE (MeSH) and Embase (Emtree) to identify key search terms. The key terms focused on marriage length and were combined using the “OR” operator (long-term marriage OR lasting marriage OR stable marriage OR sustainable marriage OR enduring marriage). The second step of the search involved the use of key search terms in a comprehensive database search without time limitation, from the inception of each database to January 30, 2019. Two reviewers independently searched the following 7 electronic databases: PubMed, Embase, Scopus, Science Direct, Web of Science, Magiran, and Scientific Information Database (SID). Two of those databases—SID and Magiran —are Persian databases. The initial literature search retrieved the following numbers of articles: MEDLINE (309), Scopus (733), Science Direct (112), Web of Science (492), Embase (56), Magiran (5), and SID (3). Overall, 1,706 articles were retrieved from the database searches, of which a total of 25 articles remained after the review process shown in Figure 1.

The inclusion criteria for articles were as follows: the participants were heterosexual couples (husbands or wives), the condition of interest was long-term marriage, the focus was on some aspect of the motivators of long-term marriage or marital satisfaction as a proxy of long-term marriage, the title and abstract of the article were written in English, and the article text was written in English or Persian.

### Data extraction

Two independent investigators extracted all major components using a pre-established structured checklist. The extracted data included study information and the participants’ characteristics (e.g., gender, location, country), as well as motivations for long-term marriage. All disagreements were discussed with the third author if necessary.

### Quality assessment

Qualitative studies were evaluated using the qualitative methodological checklist of the National Institute of Clinical Nursing (NICE). Generally, according to the NICE checklist, ++ means that all or most of the checklist criteria have been fulfilled, + means that some of the checklist criteria have been fulfilled, and – means that few or no checklist criteria have been fulfilled. Cross-sectional studies were examined using the Newcastle-Ottawa Scale for quality assessment, adapted for cross-sectional studies. This instrument is based on 3 domains, including the selection of study groups, comparability of groups, and description of exposure and outcome. All items except for the comparability domain have 1 star, and the maximum score based on stars for the comparability domain is 2. The total number of earned stars corresponds to the total quality score for each study. A cut-off score of 6 or higher was considered as indicating high-quality studies. Two review authors completed the quality assessment independently. In cases of disagreement or items that remained unclear, the third author was consulted. As shown in the quality assessment sections of Tables 1 and 2, all qualitative and most quantitative articles were of appropriate quality, and only 4 papers were of borderline-acceptable quality.

### Ethics statement

This study is a systematic review and does not deal with human participants.

## RESULTS

The articles included in the review consisted of 12 qualitative and 13 quantitative articles, of which 2 were both qualitative and quantitative. Based on the findings from the articles, the extracted factors related to long-term marriage can be classified into several main categories or dimensions. As seen in Tables 1 and 2, protective factors in marital life can be divided into intrapersonal (or intra-dyadic) and interpersonal (extra-dyadic) factors. Prominent intrapersonal factors include religiosity and spirituality, commitment and loyalty, personality characteristics, capability to trust and empathize, patience, being supportive, forgiveness, and self- and other-acceptance. Prominent interpersonal factors consist of communication, sexual relationship, love and attachment, intimacy, religious agreement, mutual respect, role division, spending quality time, and approach to problem-solving and conflict resolution. Moreover, some aspects, such as the role of children and couples’ financial issues, extend beyond the intrapersonal and interpersonal components.

As shown in Tables 1 and 2, in American countries, the most prominent aspects of couples’ relationships linked to marital stability consisted of religion, sexual relationship, commitment, intimacy, and congruence in values and beliefs. In Asian countries, the most prominent factors included communication, religion, children, conflict resolution, emotional issues and love, and in in European countries, the key factors included sexual relationship, commitment, relationship satisfaction, and support from one’s mate.

A comprehensive list of the protective factors of marital stability extracted from qualitative and quantitative articles are shown in Tables 1 and 2.

## DISCUSSION

The main finding of this study is that numerous components in many studies have been considered to be factors influencing long-term marriage stability. We divided these factors into interpersonal and intrapersonal or intra-dyadic and extra-dyadic factors. Interpersonal components refer to how behavioral interactions between members of couples are associated with relationship stability and quality, while intrapersonal components focus on how diversity in either psychopathology or personality characteristics relates to the functioning of couples’ relationships [39,40].

According to the paradigm proposed by Karney & Bradbury [21], 3 dimensions of couple’s lives affect marital stability. First, enduring vulnerabilities are aspects such as psychological disorders that affect people’s ability to interact efficiently in marital relationships. Second, stressful events include challenges such as unemployment in a couple’s daily life, and third, adaptive processes include the manners of interaction such as approaches to conflict resolution that influence a couple’s ability to successfully deal with daily stressors [41]. Therefore, we can implement the findings of the present study within this framework. Intrapersonal protective factors, such as commitment and loyalty, personality characteristics, capability to trust, and empathy can be considered as enduring vulnerabilities. Some components, such as social norms and expectations, play the role of stressful events. Next, many interpersonal factors are considered as adaptive processes, such as religious agreement and approaches to problem-solving, decision-making, and role division.

In most studies, the role of religion in long-term marriage has been highlighted. In moments of hardship, religion and spirituality are coping strategies that give couples commitment, capability to accept adversity, and a sense of family community and stability [42-46]. Similarly to the present study, numerous studies have emphasized the role of religion in marital stability and prevention of divorce [47-50]. Religious affiliation and attendance contribute to couples’ well-being, and support and foster marital relationships [51,52]. Religious couples are happier, have higher life and marital satisfaction, and have marital boundaries that preserve them in conflict situations [53,54]. Furthermore, religion teaches that marriage is sacred and divorce is to be avoided, and marriage is a place to engage with other institutions, couples, and families that support family and couple life [53,55]. When people with religious faith face difficulties, they enter into contact with God or another superior being, which gives them a sense of safety and control over the situation [56].

Commitment is defined as the desire to remain in the marriage even when confronted with confusion and difficulties [57]. Commitment theory states that couples with a powerful commitment to marriage consider marital problems to be solvable; because they believe that their paired unit can and must work to solve problems, they choose behavioral steps that improve marital relationships [58]. Therefore, we can conclude that committed couples develop an identity as a couple that gives them a sense of togetherness. Based on this, they consider themselves to be a pair with a common future, and make further efforts to preserve their identity as a couple.

Responsiveness to the positive emotions of one’s partner plays a key role in fostering stable relationships [59]. When partners experience authentication of positive, rather than negative, emotions, the possibility of accepting incongruities among partners increases [60]. Carstensen et al. [61] found that in long-term marriages, husbands were more defensive than wives, whereas wives were more emotionally negative, and that partners in happy marriages engaged more in positive emotions than those in unhappy marriages. Therefore, we can infer that positive emotional engagement between partners in a couple leads to a happy marriage, and that happiness in marriage is a protective factor of marital stability.

Sexual satisfaction is necessary for marital stability and is correlated with general happiness, mental health, and successful social communications [62]. In contrast, Blümel et al. [63] concluded that a sexual relationship is not necessary for a couple’s stability. This study also noted that good mutual understanding is a key element of a couple’s sexual relationship and that sexual satisfaction arises in happy couples. Therefore, we can state that a sexual relationship is important, but the quality of communication is paramount.

There are different ways that partners in a couple can show affection to each other, but a healthy sexual relationship is considered to be a major signifier of marital well-being and a fundamental way that couples can show care and love to each other. Thus, it may be consider as a powerful symbol of a couple’s relationship [12]. Sexual relations are an arena in which partners in a couple share love, intimacy, and deep feelings [64], creating a sense of unity and intimate belonging between couples, which leads to a diminution of individual boundaries and a strengthening of the couple’s boundaries [65].

Conflict, which refers to disagreement or incompatibility between partners in a couple, is inevitable; however, experiencing high levels of stress in conflict can be destructive of marital satisfaction and stability [66]. Based on their attachment style, partners in a couple may resolve conflict (constructive engagement), intensify conflict (destructive engagement), or avoid conflict (conflict avoidance) [67]. Of these conflict management strategies, constructive engagement is considered to be a protective factor in long-term relationships. Constructive engagement involves affirmative problem-solving approaches, which include mutual negotiation and conversation, cooperation with one’s partner, and the willing commitment to openness, spousal support, and responsibility [66]. Additionally, a link of secure attachment between partners in a couple can create a sense of support and availability, which can generate a sense of care and empathy in times when it is needed [68].

Happy couples tend to perceive constraints such as shared property, friends, and children as sources of joy and evidence of investment [58]. When partners in the role of parents cooperate as a team to care for their children, this leads to a greater appreciation of the partner and a sense of care in the relationship [69,70]; therefore, raising children creates a sense of responsibility, teamwork, togetherness, and effectiveness between partners, which can promote a stable marriage.

Regarding gender differences in perspectives on the basic protective factors of marital stability, a number of the reviewed studies reported common components for men and women, while several other studies reported different findings. A point of common consensus is that gender for men and communication for women are crucial to marital stability, but there is no agreement on other components. It seems that future research should more focus on this age-old source of disputation through questionnaires and in-depth interviews of women and men to clarify their views on this issue.

This study has some important limitations that should be considered. First, the search was conducted only in the English and Persian languages. Access to articles in other languages would have increased the breadth of the study and its social and cultural richness. This limitation may have caused us to miss a number of valuable research studies. Second, we did not search a comprehensive set of databases, so there might have been important articles in uninvestigated databases that were not included in the current study. Another limitation is that some important data, such as couples’ religion, number of children, age at first childbirth, economic status, age at the time of marriage, and status in terms of remarriage, were not investigated because few articles presented data on those factors. It is possible that information on these parameters would influence the interpretation of our findings. The final limitation is that because of inconsistency and heterogeneity in the statistical methods used in quantitative articles, it was not feasible to conduct a meta-analysis.

## CONCLUSION

It should be noted that this study identified fundamental protective factors of marital stability in long-term marriage in different countries with diverse cultures. These protective factors are inherently associated with environmental and individual differences and the interactions between them. The originality of this systematic review lies in its strength-focused perspective on protective factors of marital stability and the fact that its results can be used to educate couples on ways to strengthen the foundations of the family and for counselors and clinicians to take a preventive perspective on many preventable cases of divorce.

## Figures and Tables

**Table 1.** Protective factors of marital stability extracted from qualitative articles

Study	Study location	Sample size	Design	Marriage duration (yr)	Instrument	Motivators (marital stability factors)		QA^1^
						Themes	Sub-themes	
Timothy-Springer et al., 2016 [7]	Trinidad and Tobago	6 couples (12 participants)	Phenomenological approach	21-47	Semi-structured interview	Attitudes; action; children; approach to challenges; religion	Attitude to relationships, attitude of respect, attitude of contentment; act of role sharing, act of spending quality time	+ +
Mullins, 2016 [8]	USA	43 couples	Phenomenological approach	51 (range: 41-71)	In-depth interviews	Religion	Prayer, worship services, and sermons	+
Hatami et al., 2016 [27]	Iran	10 couples	Grounded theory	20 or more	Semi-structured interviews	Intrapersonal factors	Self- acceptance, spouse acceptance, positive attitude to families of origin, expecting to have problems in life	+ +
						Interpersonal factors	Religious alignment, forgiveness, love and attachment, good sexual relationship, raising children	
						Meta-personal factors	Having a stable job, not having severe financial problems	
Elliott et al., 2008 [12]	USA	31 couples	Grounded theory	8-52 (mean: 25)	In-depth interviews	Sexual intimacy	-	+ +
Jeffries, 2006 [9]	USA	49 couples	Quantitative and qualitative	25 or more	Unstructured interviews and questionnaire	Benevolent love	-	+
Mackey et al., 2005 [10]	USA	144 couples	Quantitative and qualitative	Over 20 (mean: 35.25)	In-depth interviews	Religion	Emerging spirituality	+ +
Hinchliff et al., 2004 [28]	UK	28 participants	N/A	43 (range: 22-61)	Semi-structured interview	Sexual relationship	-	+ +
Bachand et al., 2001 [2]	Maine (USA)	15 couples	Phenomenological approach	35-54 (mean: 43.2)	Interviews	Friendship; love; similar interests; commitment; freedom to pursue goals; knowing one another well before; respect for other; similar values; spouse is a good person; conscious of other’s feelings; support; tolerance; religion/religious agreement; partnership; trust; children; communication; loyalty; patience; companionship; compromise; forgiveness; put the other first	-	+ +
Tilse, 1994 [15]	Australia	18 participants	Qualitative approach	10 or more	In-depth interviews	Living together; couple identity; obligation to care for each other; attachment and loyalty	-	+
Robinson, 1994 [29]	USA	15 couples	N/A	At least 30	Interviews	Religious faith	Emotional support, social support, spiritual support, heterogamy	+ +
Robinson et al., 1993 [30]	USA	15 couples	Phenomenological approach	35-48 (mean: 40.4)	Unstructured interviews	Intimacy; commitment; communication; congruence; religious faith	-	+ +
Roberts, 1979-1980 [31]	Arizona (USA)	50 couples	N/A	55.5 (range: 50-65)	Structured interviews	Life without dependence on others; agreement in decision-making; sexual attitudes; commitment; companionship; qualities of caring	-	+

QA, quality assessment; N/A, not available.

1 According to the National Institute of Clinical Nursing checklist, ++ means that all or most of the checklist criteria have been fulfilled, + means that some of the checklist criteria have been fulfilled.

**Table 2.** Protective factors of marital stability extracted from quantitative articles

Study	Study location	Sample size	Design	Marriage duration (yr)	Instrument	Motivators (marital stability factors)	Measure of association	Values	QA^1^
Koraei et al., 2017 [13]	Iran	239 woman	Analysis of covariance matrix correlation	15 or more	Questionnaire	Conflict resolution	Pearson correlation between factors and Hendrik Relationship Assessment Scale	0.76	7
						Protection		0.75	
						Responsibility		0.66	
						Quality of sex		0.69	
						Quality of marital life		0.63	
						Couple congruence		0.68	
						Commitment		0.64	
						Shared values		0.54	
						Financial and business issues		0.52	
Landis et al., 2013 [14]	Germany and Switzerland	132 couples	Correlation	42 (range: 25-57)	Questionnaire	Dyadic coping (relation with relationship satisfaction)	Pearson correlation	Wives: 0.28	7
								Husbands: 0.23	
Phillips et al., 2012 [11]	USA	71 couples	Descriptive	32 (range: 15-60)	Questionnaire (open-ended questions)	God/Jesus	Frequencies (%)	51	5
						Love		31	
						Good communication		23	
						Honesty		15	
						Shared religious beliefs		13	
						Have remained friends		13	
						Commitment		11	
						Respect		10	
Pnina, 2009 [16]	Israel	128 couples	Correlational	More than 45	Questionnaire	Role division	t-test (first 3 years and present)	Wives:-5.05	8
								Husbands: 3.16	
Jeffries, 2006 [9]	USA	49 couples	Quantitative and qualitative	25 or more	Unstructured interview and questionnaire	Religious beliefs	Pearson correlation	0.34	5
						Manifestation of love		0.40	
						Receiving benevolent love		0.73	
Mackey et al., 2005 [10]	USA	144 couples	Quantitative and qualitative	Over 20 (mean: 35.25)	In-depth interviews	Religion	Logistic regression	Beta=0.4	6
								Exp(B)=1.5	
Hatch et al., 2004 [22]	USA	Cross-sectional (5,448)	Cross-sectional and longitudinal	10 or more	Questionnaire	Less frequent disagreements	Regression	Time×age/cohort (0.44)	7
		Longitudinal (4,401)					General linear model	Time×marital duration (2.63)	
Goodman, 1999 [32]	USA	180 participants	N/A	25 or more	Questionnaire	Intimacy was a positive predictor and hostile control was a negative predictor	Multiple regression	Hostile control: β=-0.382	8
								Intimacy: β=0.0431	
Roizblatt et al., 1999 [33]	Chilean segment of a multicultural (Canada, Germany, Israel, Netherlands, South Africa, Sweden, USA)	56 couples	N/A	More than 25	Questionnaire	Values and beliefs	Frequencies (%)	N (56.0)/Ds (48.0)	5
						Intrinsic motivation		N (53.0)/Ds (58.0)	
						Mutuality		N (45.0)/Ds (56.0)	
						Extrinsic motivation		N (39.0)/Ds (37.0)	
						Social norms and expectation		N (38.0)/Ds (35.0)	
						Positive problem-solving		N (35.0)/Ds (30.0)	
Sharlin, 1996 [34]	Israel	50 couples	N/A	34 (25-40)	Questionnaire	Love; enjoy lifestyle; marriage is a partnership for life; experiences have drawn us so closely together; we appreciate our closeness	Frequencies	N/A	7
Lauer et al., 1990 [24]	USA	100 couples	N/A	45 or more	Questionnaire	Mate is best friend; enjoyed together; long-term commitment; marriage is sacred; agree on aims and goals; laugh together; proud of mate’s achievements; mate more interesting now than when married; outside interests; agree on major decisions; expression of affection; agree on philosophy of life	Frequencies	Spearman correlation coefficient with Lauer and Lauer (1986) for the 10 most frequently named reasons was 0.79	6
Lauer et al., 1986 [35]	USA	351 couples	N/A	15 or more	In-depth interviews and questionnaire	Mate is best friend	Frequencies	188	5
Swensen et al., 1985 [36]	Norway	72 subjects	N/A	37.3	Interviews and questionnaire	Commitment	t-test for differences between commitment at start of marriage and the present time	3.63	6
Fields, 1983 [37]	USA	290 participants (145 couples)	Correlational	18-30	Questionnaire and interviews	Congruence of perceptions	Pearson r correlations (with marital satisfaction)	Women: 0.43	7
								Men: 0.28	
Ard, 1977 [38]	USA	161 couples	N/A	20 or more	Questionnaire	Sexual relationship	Frequencies and t-test for difference (between husbands and wives)	95% of husbands and 90% of wives; t=6.33	6

QA, quality assessment; N/A, not available; N, now; Ds, difficult states.

1 Newcastle-Ottawa Scale marking for quality assessment of cross-sectional studies.
